# Synchrotron- and focal plane array-based Fourier-transform infrared spectroscopy differentiates the basalis and functionalis epithelial endometrial regions and identifies putative stem cell regions of human endometrial glands

**DOI:** 10.1007/s00216-018-1111-x

**Published:** 2018-05-09

**Authors:** Georgios Theophilou, Camilo L. M. Morais, Diane E. Halliwell, Kássio M. G. Lima, Josephine Drury, Pierre L. Martin-Hirsch, Helen F. Stringfellow, Dharani K. Hapangama, Francis L. Martin

**Affiliations:** 1 Department of Gynaecology, Leeds Teaching Hospitals NHS Foundation Trust, Leeds, LS1 3EX UK; 2 School of Pharmacy and Biomedical Sciences, University of Central Lancashire, Preston, PR1 2HE UK; 3 Biological Chemistry and Chemometrics, Institute of Chemistry, Federal University of Rio Grande do Norte, Natal, 59072-970 Brazil; 4 Department of Obstetrics and Gynaecology, Liverpool Women’s NHS Foundation Trust, Liverpool, L8 7SS UK; 5 Department of Obstetrics and Gynaecology, Royal Preston Hospital, Lancashire Teaching Hospitals NHS Foundation Trust, Fulwood, Preston, PR2 9HT UK

**Keywords:** Biospectroscopy, Cell lineage, Chemometrics, Endometrium, FTIR spectroscopy, Stem cells

## Abstract

The cyclical process of regeneration of the endometrium suggests that it may contain a cell population that can provide daughter cells with high proliferative potential. These cell lineages are clinically significant as they may represent clonogenic cells that may also be involved in tumourigenesis as well as endometriotic lesion development. To determine whether the putative stem cell location within human uterine tissue can be derived using vibrational spectroscopy techniques, normal endometrial tissue was interrogated by two spectroscopic techniques. Paraffin-embedded uterine tissues containing endometrial glands were sectioned to 10-μm-thick parallel tissue sections and were floated onto BaF_2_ slides for synchrotron radiation-based Fourier-transform infrared (SR-FTIR) microspectroscopy and globar focal plane array-based FTIR spectroscopy. Different spectral characteristics were identified depending on the location of the glands examined. The resulting infrared spectra were subjected to multivariate analysis to determine associated biophysical differences along the length of longitudinal and crosscut gland sections. Comparison of the epithelial cellular layer of transverse gland sections revealed alterations indicating the presence of putative transient-amplifying-like cells in the basalis and mitotic cells in the functionalis. SR-FTIR microspectroscopy of the base of the endometrial glands identified the location where putative stem cells may reside at the same time pointing towards ν_s_PO_2_^−^ in DNA and RNA, nucleic acids and amide I and II vibrations as major discriminating factors. This study supports the view that vibration spectroscopy technologies are a powerful adjunct to our understanding of the stem cell biology of endometrial tissue.

**Electronic supplementary material:**

The online version of this article (10.1007/s00216-018-1111-x) contains supplementary material, which is available to authorized users.

**Graphical abstract:**
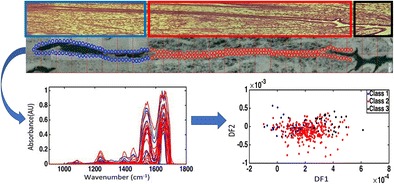
ᅟ

## Introduction

The endometrium is a highly regenerative tissue that undergoes monthly cycles of shedding and regeneration under the influence of oestrogen and progesterone [1, 2]. It comprises two regions, the functionalis and the basalis. The functionalis is the region that is shed with menstruation and is composed of glands lined by pseudostratified columnar epithelium during the proliferative phase of the menstrual cycle and columnar epithelium in the secretory phase. It is surrounded by vascularised stroma. The basalis comprises of the bases of the glands, surrounded by vascular endothelium and denser stroma [3]. The basalis layer extends into the myometrium. The endometrial/myometrial junction is the transition zone between the glandular epithelium and the stroma of the endometrium and the inner myometrium, which are not separated by a submucosal layer. Despite this, they are clearly distinguishable by light microscopy. Embryologically, these areas are of similar origin arising from the paramesonephric ducts, while the outer myometrium is thought to be of non-paramesonephric origin.

Adult stem cells are rare undifferentiated cells that function to maintain tissue homeostasis by generating replacement cells for tissues during routine cellular turnover or for the repair of tissues during injury [4]. Their defining properties are proliferation and self-renewal and differentiation into multiple lineages depending on the tissues they aim to regenerate [5]. They reside amongst niche cells and extracellular matrix which transmit signals that regulate their activity, at the same time guarding their genetic stability [4]. In the endometrium, there are three types of stem cells, namely epithelial, endothelial and mesenchymal stem cells [6]. It has been hypothesised that stem cells reside within the epithelial layer lining the glands of the basalis layer as well as the surrounding stroma and are responsible for the cyclical regeneration of the functionalis layer [1]. At the present time, there are no conclusive markers to isolate epithelial progenitor cells in menstrual blood while stromal progenitor cells have been identified [7]. This suggests that the former may reside exclusively in the luminal epithelium of the basalis layer [8]. Their daughter cells are termed transient-amplifying cells and migrate to the functionalis layer resulting in its proliferation. Several techniques have been exploited to confirm the presence, location and activity of endometrial stem cells. These include cloning studies [9, 10], immunochemistry studies [11, 12] and regeneration studies [13]. Expression of immunochemical markers for such stem cells was demonstrated in human endometrium, but the exact location of most of these cells has not been pinpointed [14]. The SSEA1 expressing epithelial cells from basalis glands have shown some progenitor activity *in vitro* [12], but their abundance suggests that only a subpopulation of SSEA1 expressing epithelial cells from basalis glands has adult stem cell properties [12].

Stem cells may be implicated in uterine carcinogenesis. Cancer stem cells possess similar properties to stem cells in terms of differentiation and self-renewal potential. They differ from benign adult stem cells in that their growth potential is no longer controlled by signals from the surrounding niche cells; therefore, they proliferate uncontrollably and differentiate into unpredictable cellular lineages [15]. Cancer stem cells may be derived from resident adult stem cells through genetic or epigenetic changes [15].

Endometrial stem cells may also be implicated in the pathogenesis of endometriosis. Sampson’s retrograde menstruation theory states that endometrial fragments that enter the peritoneal cavity through the fallopian tubes during menstruation implant into peritoneal surfaces and undergo similar cycles of proliferation and shedding as normal endometrium. It is hypothesised that these fragments may contain endometrial stem cells that are abnormally shed during menses and have the ability to implant into ectopic surfaces and proliferate forming endometriotic lesions [16, 17]. Although endometrial stem cells have been identified in menstrual blood, they have not been recognised in peritoneal fluid in higher quantities during menstruation [18]. This may be due to the lack of structural markers for these cells.

Infrared (IR) spectroscopy is a powerful technique to investigate biological tissues, since it can detect many important biochemical signatures including amide I (~ 1650 cm^−1^), amide II (~ 1550 cm^−1^), protein (~ 1425 cm^−1^), amide III (~ 1260 cm^−1^), asymmetric phosphate stretching vibrations (ν_as_PO_2_^−^; ~ 1225 cm^−1^), carbohydrates (~ 1155 cm^−1^), symmetric phosphate stretching vibrations (ν_s_PO_2_^−^; ~ 1080 cm^−1^) and protein phosphorylation (~ 970 cm^−1^) [19]. Successful applications of IR spectroscopy towards the analysis of tissue samples include cancer identification in breast, lung, colon and prostate tissues [20, 21]. Endometrial tissues have been also investigated using IR spectroscopy, where results for differentiating benign and malignant tissues were mainly assigned to lipid and amide I/II regions [22]. Putative stem cells have been studied in human intestinal crypts using IR spectroscopy where ν_s_PO_2_^−^ were found to be the main biomarker for distinguishing different putative cell types [23].

Amongst different types of IR techniques, synchrotron radiation-based Fourier-transform infrared (SR-FTIR) and focal plane array (FPA) FTIR spectroscopy are excellent techniques for investigating tissue samples [21]. In SR-FTIR, a synchrotron source emits a collimated light beam more intense than that of a bench-top spectrometer. This provides an excellent signal-to-noise ratio (SNR) that is 1000 times greater to that of conventional IR sources and allows spatial resolutions as small as 10 μm [24, 25]. The absence of thermal noise and the order of intensity magnitude greater for synchrotron radiation source in infrared microspectroscopy increased the SNR performance in comparison to operation with a built-in globar (thermal) source, therefore generating a spectra with higher resolution than regular FTIR [21]. On the other hand, FPA uses an IR focal plane array detector to generate hyperspectral imaging. The multiple detector elements of the FPA detector enable the concurrent acquisition of several spectra at each spatial point of the area of the sample under investigation. This allows the examination of larger areas at reasonable experimental time periods. This method results in the formation of a “hypercube” which contains information in two spatial dimensions: a pseudo-image and one spectral dimension, corresponding to the spectrum for each point (pixel) of that image [21].

Biospectroscopy is a powerful technology that can be used in conjunction to other biomarker-based isolation techniques to attempt the identification of endometrial stem cells amongst their progeny. The characterisation of endometrial stem cells with regard to their molecular, genetic and epigenetic make-up may assist in unravelling their role in many endometrial proliferative disorders such as endometriosis, adenomyosis and carcinogenesis. In this paper, SR-FTIR and FPA-FTIR microspectroscopy were used to investigate endometrial tissue samples. SR-FTIR microspectroscopy was used for identification of putative stem cells amongst the epithelial cells on the basalis portion of endometrial glands, while FPA-FTIR microspectroscopy was used to distinguish epithelial cells lining in the basalis portion from those lining in the functionalis portion of the endometrial glands.

## Methods

### Tissue collection and preparation

Ethical approval was obtained from Liverpool Adult Research Ethics Committee (LREC 09/H1005/55 and 11/H1005/4), and the study was conducted according to the principles of the Declaration of Helsinki and all other applicable national or local laws and regulations. Written, informed consent was obtained from all participants prior to inclusion in the study at the Liverpool Women’s NHS Foundation Trust.

Three secretory phase pre-menopausal uteruses from women undergoing hysterectomy for non-endometrial benign causes (uterine prolapse) who had not been on hormonal treatments in the preceding 3 months were collected. A wedge of tissue from the lumen to the muscular myometrial layer that included superficial and basal endometrium as well as myometrium was taken from the detached uterus, collected in normal buffered formalin and subsequently embedded in paraffin. Several 10-μm-thick parallel tissue sections were cut from each sample and floated onto 1 cm × 1 cm BaF_2_ slides (Photox Optical Systems). These were de-waxed by serial immersion in three sequential fresh xylene baths for 5 min and washed in an acetone bath for a further 5 min. The resulting samples were allowed to air dry and placed in a desiccator until analysis. Four-micrometre-thick parallel tissue sections were floated to glass slides and stained with haematoxylin and eosin (H&E) for histological comparison.

### Synchrotron radiation-based Fourier-transform infrared microspectroscopy

**Fig. 1:**
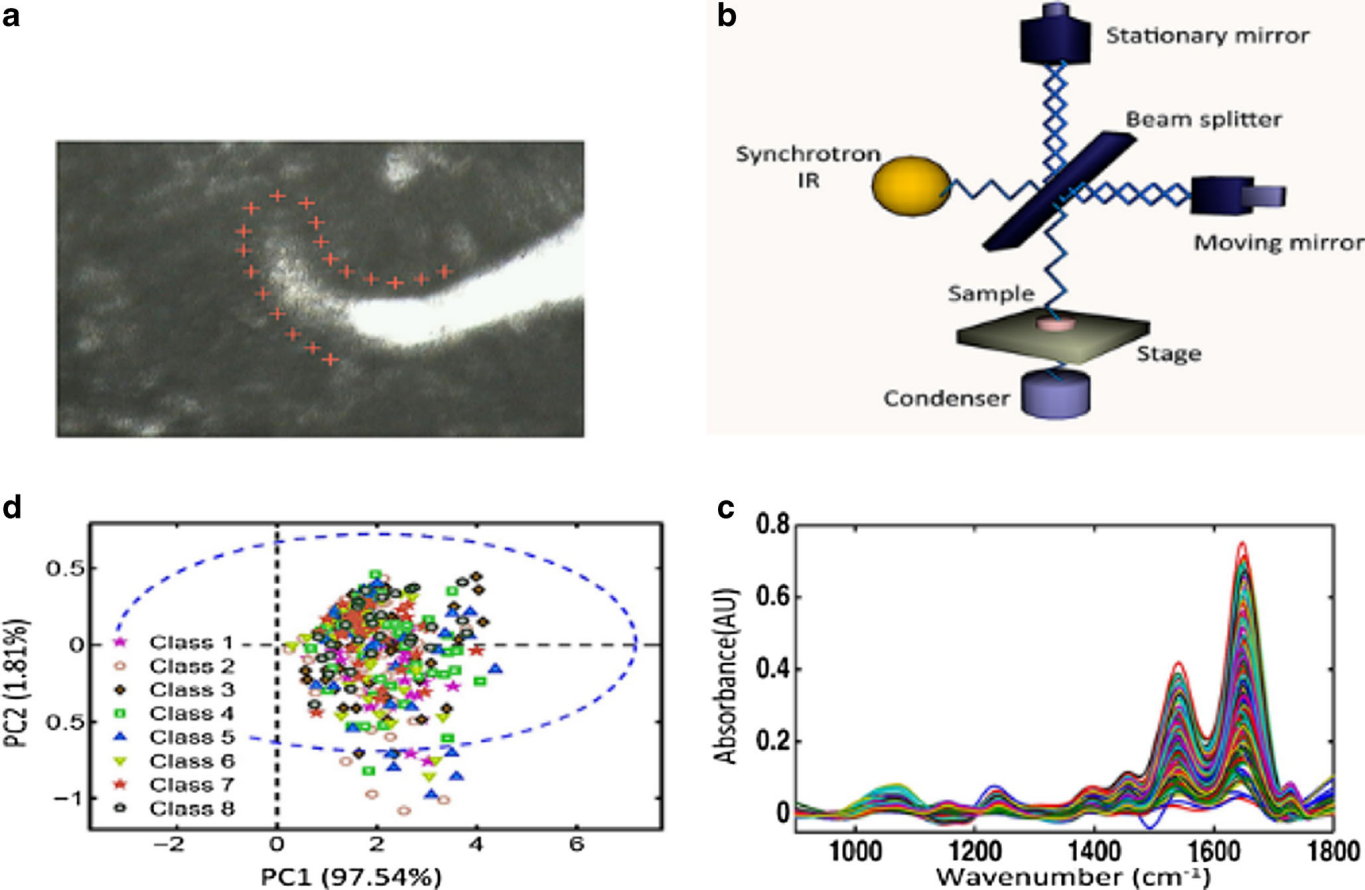
Synchrotron radiation-based FTIR spectral interrogation and analysis of endometrial epithelial gland bases to identify the location of putative stem cells. **a** Micrograph with superimposed marks representing areas interrogated by synchrotron radiation (magnification ×36). **b** Diagram representing a Michelson interferometer, which is the basic structure of an IR spectroscope. **c** The interferogram is transformed to an absorbance spectrum by Fourier-transform. **d** PCA identifies “outliers” which may represent putative stem cells

The SR-FTIR microspectroscopy setup is illustrated in Fig. 1. Spectral mapping data from 4000 to 600 cm^−1^ were obtained at the Diamond Light Source Ltd., UK (www.diamond.ac.uk) at beamline 22IR. Optically interfaced to the beamline were a Bruker Vertex 80v FTIR spectrometer, a Bruker Hyperion 3000 microscope equipped with front surface reflecting optics, a liquid nitrogen mercury cadmium telluride (MCT) detector and a ×36 objective lens. Spectra were collected in transmission mode from specimens onto BaF_2_ slides. At 10-μm step sizes, 256 spectra were co-added with the aperture set for 10 μm × 10 μm (numerical aperture [NA] = 0.50, lateral resolution 6.7–12.2 μm). Mapping data were acquired within 6 h (Fig. 1a) with background spectra updated after every 10 spectra. Spectra recorded in transmittance were converted and displayed as absorbance with the internal Bruker Optics OPUS 8 software (Bruker Optics, Ettlingen, Germany).

### Thermal source focal plane array Fourier-transform infrared microspectroscopy

**Fig. 2:**
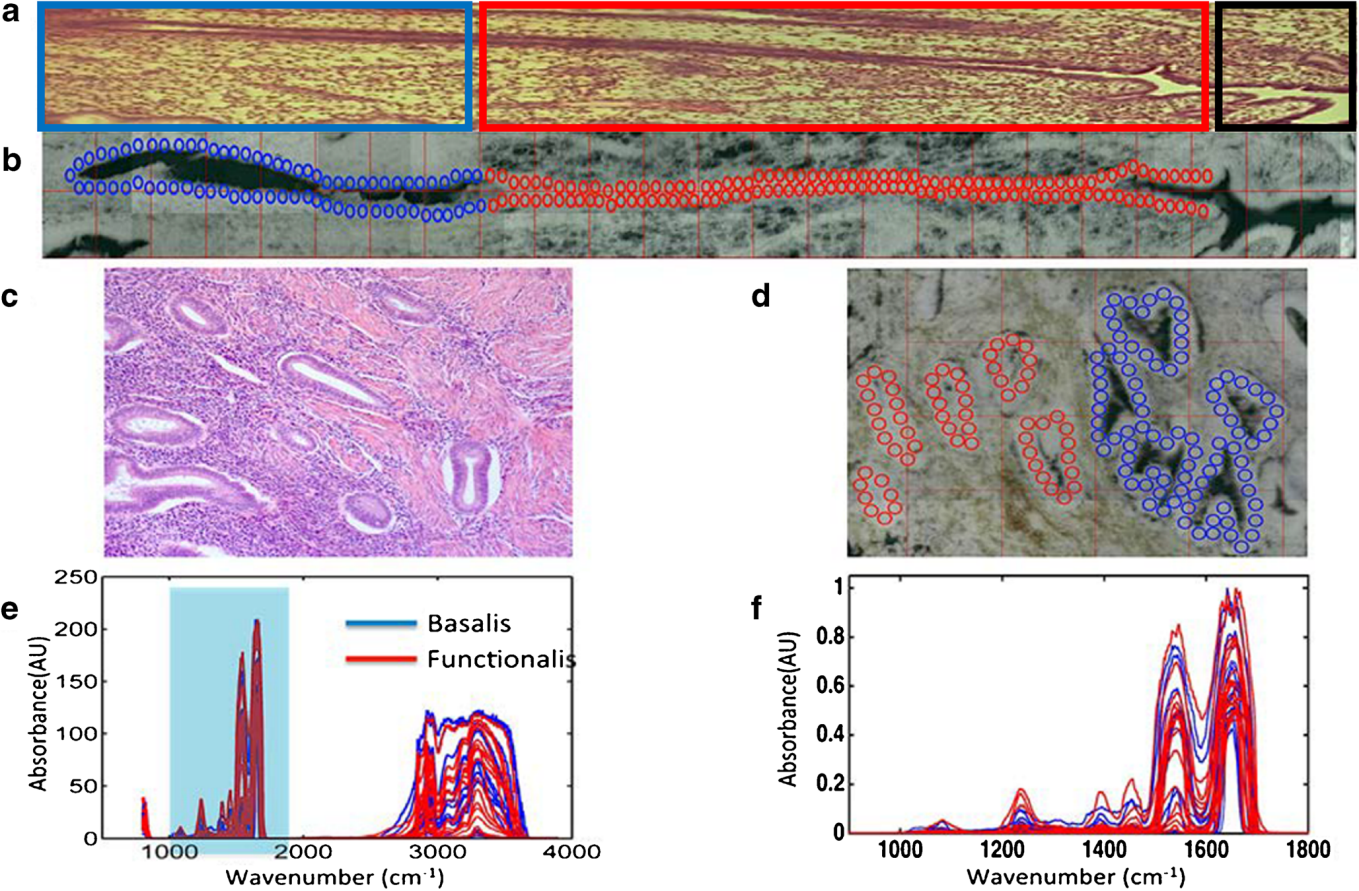
Representation of region identification for spectral selection by FPA and pre-processing of acquired spectra. **a** H&E-stained parallel section of the examined longitudinally cut crypt, in the secretory phase (circled in blue: cells in the deepest portion of the gland; circled in red: basalis/functionalis cells; circled in black: cells close to the gland opening into the endometrial cavity). **b** Micrograph of the examined longitudinally cut crypt with overlaid circular markers representing epithelial areas which were selected for comparison (magnification ×15) (the actual spectral area selection on the pseudo-image was performed at different magnifications to ensure the spectra selected were good representatives of the areas to be examined). **c** H&E-stained parallel section of the examined coronally cut crypt, in the secretory phase. **d** Micrograph of the examined coronally cut crypt with overlaid circular markers representing epithelial areas which were selected for comparison (magnification ×15) (the actual spectral area selection on the pseudo-image was performed at different magnifications to ensure the spectra selected were good representatives of the areas to be examined). **e** Averaged absorbance spectra acquired by FPA imaging of all samples. **f** Pre-processed spectra resulting from the raw spectra acquired by FPA imaging of all samples

A Bruker Vertex 80v FTIR spectrometer and Hyperion 3000 microscope were employed using the built-in globar (thermal) infrared source. A 64 × 64 cooled photodiode FPA collected spectra from 4000 to 600 cm^−1^ in parallel at 4 cm^−1^ resolution (NA = 0.65, lateral resolution 5.2–9.4 μm). Co-addition of 128 sample scans and 256 background scans was used. Matching ×15 Cassegrain objective and condenser mirror lenses were used for transmission measurement of microtomed tissue sections mounted on BaF_2_ slides (1 cm × 1 cm). FPA imaging maps were acquired from several sections from the three uterine samples involving basalis/functionalis junctions containing epithelial glands. The fidelity of the FPA spectral image region between 1800 and 900 cm^−1^ at 4 cm^−1^ spectral resolution (468 wavenumbers with data spacing of 1.9 cm^−1^) is the area associated with the biological spectral fingerprint [26] (Fig. 2).

### Data pre-processing

Absorbance spectral images were converted to suitable digital files (.txt) for analysis within MATLAB R2014a environment (MathWorks Inc., Natick, USA). They included wavenumber regions scanned between 4000 and 600 cm^−1^ that were truncated to include only the fingerprint region between 1800 and 900 cm^−1^. The resulting dataset was smoothed by Savitzky-Golay filter, rubber band baseline-corrected and normalised to the amide I peak (i.e. ≈ 1650 cm^−1^) [21, 27]. The normalisation to amide I peak is commonly performed after baseline correction in order to correct spectra having different absorbance values at amide I region [21]. This prevents the effect of samples having different thicknesses or concentrations that can mask the actual biochemical difference of interest [27]. The importing and pre-treatment of the spectral data and the construction of chemometric classification models were performed using PLS Toolbox 7.8 (Eigenvector Research Inc., Wenatchee, USA) within MATLAB software.

### Computational analysis

Computational analysis consisted of three models: principal component analysis (PCA), successive projections algorithm (SPA) and genetic algorithm (GA). All models were followed by linear discriminant analysis (LDA) [28]. Before applying each analytical model, spectral data were divided into training (70%), validation (15%) and prediction (15%) sets by applying the classic Kennard-Stone (KS) uniform sampling algorithm [29]. The training set was dependent on the number of pixels of the FPA detector for the same IR source, whereas the prediction set was only used for the final classification evaluation.

PCA is a multivariate analysis technique that aims to reduce the number of variables present in the spectral dataset [30]. Principal components (PCs) capture most of the variance present in the original dataset. After PCA decomposition, the original data is represented by a combination of scores and loadings. The scores represent the variance on the samples’ direction; therefore, they show the similarities and dissimilarities amongst the samples; and the loadings represent the variance on the variables direction; therefore, they show the weight of each wavenumber for the pattern observed on the scores. The scores are used as input for LDA discriminant analysis in PCA-LDA [31]. In addition, after PCA, it is possible to obtain the Q residual value calculated as the sum of the squares of the residual values at each variable for each sample (squared prediction error). This indicates the similarity of the residuals between the samples and its projection into the principal component space, representing the lack of fit in the PCA model. This is therefore a tool for outlier detection [32].

SPA is a forward feature selection method [33], which operates solving co-linearity problems by selecting wavelengths whose information content is minimally redundant. The model is built through a series of interactions, starting with one wavelength and then incorporating a new one at each iteration until a specified number (*N*) of wavelengths is reached. Different from PCA, SPA does not modify the original data space, since the projections are used only for selection purposes; thus, the relationship between spectral variables and the original data space is preserved [34].

Genetic algorithms are combinational algorithms inspired by Mendelian genetics. They use a combination of selection, recombination and mutation to evolve a solution to a problem. They treat data as chromosomes allocating reproductive opportunities in such a way that those chromosomes, which represent a better solution to the target problem, are given more chances to “reproduce” than those which represent poorer solutions [34, 35]. The GA routine was carried out using 100 generations containing 200 chromosomes each. Crossover and mutation probabilities were set to 60 and 10%, respectively. Moreover, the algorithm was repeated three times, starting from different random initial populations. The best solution, in terms of the fitness value, resulting from the three realisations of the GA was employed.

LDA was performed following the application of each of the analytical models. LDA scores, loadings and discriminant function (DF) values were obtained. Usually, the first LDA factor is used to visualise the main biochemical alterations within the sample on a 1-D scores plot. The optimum number of variables for SPA-LDA and GA-LDA was determined by the minimum cost function *G* calculated for a given validation dataset as:

1 $$ G=\frac{1}{N_{\mathrm{V}}}{\sum}_{n=1}^{N_{\mathrm{V}}}{g}_n $$

where *N*_V_ is the number of validation samples and *g*_*n*_ is defined as:

2 $$ {g}_n=\frac{r^2\left({x}_n,{m}_{I(n)}\right)}{\min_{I(m)\ne I(n)}{r}^2\left({x}_n,{m}_{I(m)}\right)} $$

in which *r*^2^(*x*_*n*_, *m*_*I*(*n*)_) is the squared Mahalanobis distance between object *x*_*n*_ (of class index *I*(*n*)) and the centre of its true class (*m*_*I*(*n*)_), and *r*^2^(*x*_*n*_, *m*_*I*(*m*)_) is the squared Mahalanobis distance between object *x*_*n*_ and the centre of the closest wrong class (*m*_*I*(*m*)_) [36].

For each model, sensitivity (the confidence in a positive result for a sample of the label class is obtained) and specificity (the confidence that a negative result for a sample of the non-label class is obtained) were calculated as important quality standards in test evaluation. The quality metrics used in this study for evaluating the classification results can be calculated following the equations [36]:

3 $$ \mathrm{Sensitivity}\left(\%\right)=\frac{\mathrm{TP}}{\mathrm{TP}+\mathrm{FN}}\times 100 $$

4 $$ \mathrm{Specificity}\left(\%\right)=\frac{\mathrm{TN}}{\mathrm{TN}+\mathrm{FP}}\times 100 $$

where FN is defined as false negative, FP as false positive, TP as true positive and TN as true negative.

## Results

### Differences between basalis and functionalis for all specimens by FPA imaging

In order to investigate the presence of spectral differences between the functionalis and basalis epithelial layers, all specimens were interrogated using FPA imaging. A raster scan approach was applied to include the epithelial layers surrounding the glandular base within the specimens. Micrographs of the involved areas with overlaid markers were used for identification of the specific epithelial regions from which spectra were acquired (Fig. 2b, d). Visual correlation with parallel H&E tissue sections was used for selection of different epithelial regions (Fig. 2a, c). Chemometric analyses of the location-derived spectra (Fig. 2e) allowed classification into basalis and functionalis regions.

**Fig. 3:**
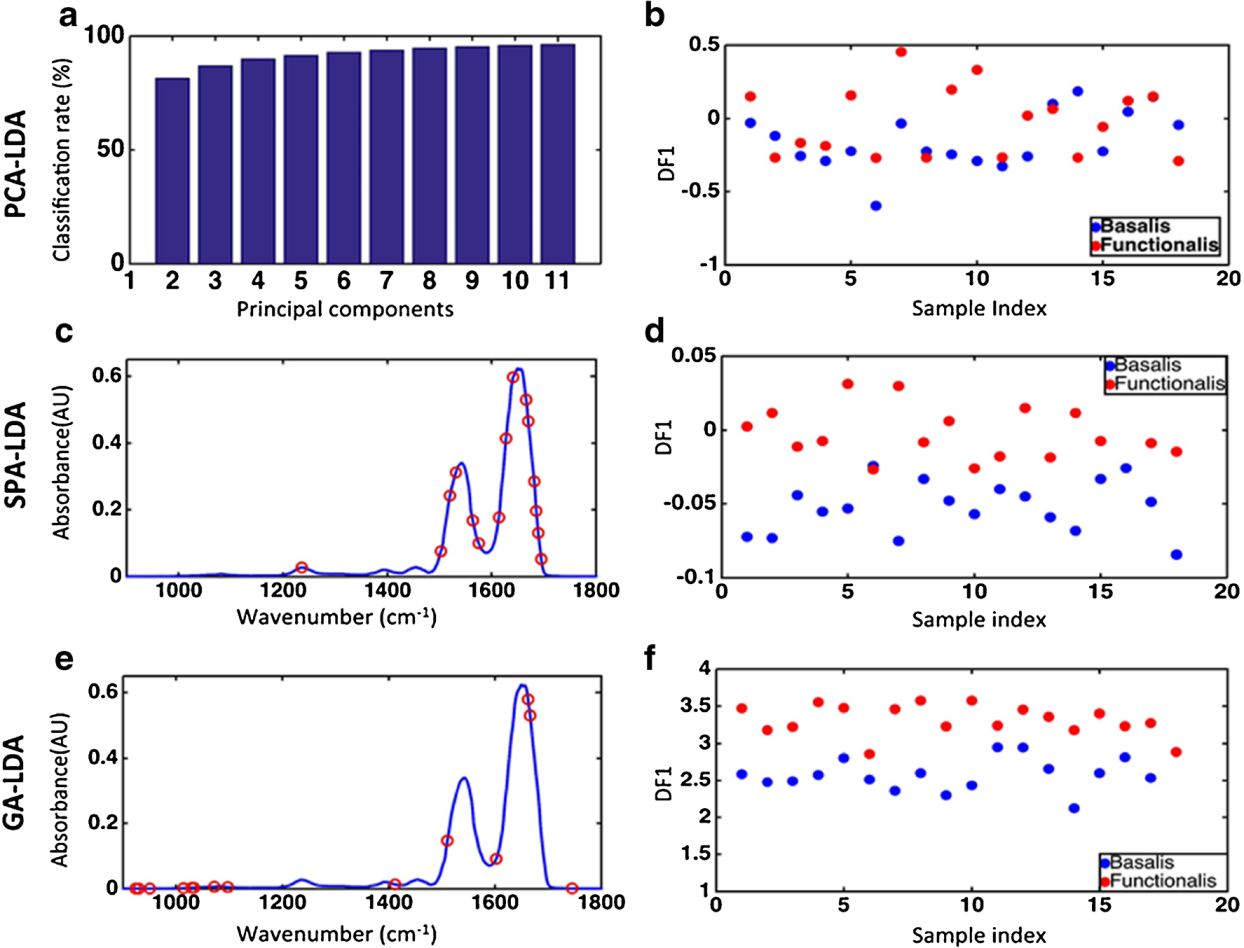
Classification of basalis and functionalis regions by spectral analysis using PCA-LDA, SPA-LDA and GA-LDA on FPA-FTIR derived data (red = functionalis, blue = basalis): All specimens. **a** Cost/function plot identifying the optimal number of PCs to be used for PCA. **b** Scores plot graphically representing classification by PCA-LDA. The *x*-axis represents the sample index and the *y*-axis the discriminant factor 1 (DF1). **c** Wavenumber selection for SPA-LDA. **d** Scores plot graphically representing classification by SPA-LDA. The *x*-axis represents the sample index and the *y*-axis DF1. **e** Wavenumber selection for GA-LDA. **f** Scores plot graphically representing classification by GA-LDA. The *x*-axis represents sample index and the *y*-axis DF1

The chemometric analyses employed for the differentiation of the epithelial cells residing in the two regions included PCA-LDA, SPA-LDA and GA-LDA that were applied to the pre-processed dataset. In total, *n* = 65,832 spectra resided within the functionalis class, and *n* = 38,357 spectra within the basalis class for the 18 areas were interrogated for comparison. Figure 2f shows the pre-processed spectra with the basalis area depicted in blue and the functionalis area in red. Overall, the IR spectra appear to have similarities in the biochemical fingerprint region (1800 to 900 cm^−1^). Figure 3a shows the explained variance for each PC using PCA-LDA. Eight PCs (explained variance = 89.41%) were used for model construction. Figure 3b shows the 1-D scores plot derived by PCA-LDA. It reveals some segregation of the basalis from the functionalis, although with some overlap of scores along DF1. The majority of the difference between the two regions was attributed to absorptions at 1540 and 1625–1675 cm^−1^ according to the PCA loadings [see Electronic supplementary material (ESM) Fig. S1a].

Although GA-LDA classified very successfully the two classes using 14 variables determined from the minimum cost function *G* (Fig. 3e) (see ESM Table S2), in which the related 1-D scores plot from different classes dissociated along DF1 is shown in Fig. 3f, the chemometric technique that classified the two classes most successfully was SPA-LDA using 12 variables (Fig. 3c) (see ESM Table S2), since better classifying performance was achieved using this technique in the prediction set (Table 1). Also, the related 1-D scores plot illustrates that spectral points from different classes dissociate very well along DF1 (Fig. 3d).

**Table 1:** Predictive performance presented as sensitivity and specificity rates calculated for each chemometric model developed to differentiate basalis and functionalis tissues from the FPA-FTIR spectral data

	PCA-LDA	SPA-LDA	GA-LDA
Basalis			
Sensitivity (%)	66.7	100.0	75.0
Specificity (%)	66.7	100.0	100.0
Functionalis			
Sensitivity (%)	0.0	100.0	60.0
Specificity (%)	0.0	100.0	100.0

All three techniques identified differences that aided classification within similar spectral regions. These differences were tentatively identified in the spectral regions of 1650 cm^−1^ (amide I band) and 1510 cm^−1^ (C-N stretch contribution to the amide II vibrational band). The overall classification rates by each chemometric technique employed to differentiate the basalis from the functionalis using FPA spectral data are shown in ESM Table S3.

### Differences between basalis and functionalis in different individuals by FPA imaging

To investigate whether similar spectral areas are able to identify segregation between the basalis and functionalis regions in different individuals, the same chemometric methods were applied on each of the specimens separately. We used the same fingerprint spectral regions for each of the specimens (see ESM Figs. 2a, 3a and 4a). When the average values of the spectra derived from the basalis layer were compared with those from the functionalis layers, subtle differences were visualised (see ESM Figs. S2b, S3b and S4b). The classification rates produced by the different chemometric techniques (see ESM Table S4) varied from 50.0 to 100.0% in the prediction set. The wavenumbers used for SPA-LDA and GA-LDA in this case are presented in ESM Table S5 and graphically in ESM Figs. S2g and S2e, S3g and S3e, and S4 g and S4e, respectively. PCA loadings for these specimens are shown in ESM Fig. S1b, c and d, in which it is observed that most of the differences between the two regions are associated with absorptions on 1235, 1550 and 1628–1685 cm^−1^. Associated scores plots graphically represent the separation identified between the two layers utilising the three chemometric techniques (see ESM Figs. S2, S3 and S4: d, f and h). Similar spectral areas seem to be causing segregation of the two histologic regions when specimens are analysed separately. These areas mostly include the spectral regions of 1650 cm^−1^ (amide I band) and 1510 cm^−1^ (C-N stretch contribution to the amide II vibrational band). These spectral areas correspond to those responsible for class segregation when all specimens were examined together. This means that spectral variations causing segregation of the two histological areas are constant independent of other inter-individual variability that may exist within the sample pool.

### Inter-individual differences within the basalis layer by FPA imaging

To investigate if the basalis layer is dissimilar in different individuals, PCA-LDA, SPA-LDA and GA-LDA approaches were applied to the pre-processed spectra derived from the basalis regions of all specimens (see ESM Fig. S5a). ESM Fig. S5b shows the average spectrum taken from the basalis layer of each individual. The 2-D scores plots in ESM Fig. S5d, f and h all show clustering of spectra from each individual as well as segregation of the spectra from different specimens. PCA-LDA identified eight PCs (explained variance = 92.61%) as the main responsible for this variation (ESM Fig. S5c). SPA-LDA utilised 12 wavenumbers (ESM Fig. S5e) and GA-LDA utilised 11 wavenumbers (ESM Fig. S5g). The classification rates between the specimens within the basalis layer are shown in ESM Table S6. ESM Table S7 shows the wavenumbers used for the analyses using SPA-LDA and GA-LDA. Wavenumbers between 1600 and 1700 cm^−1^ are consistently responsible for the segregation between the basalis layers of different specimens as well as for the classification into basalis and functionalis layers as shown above.

### Investigation of epithelial variability at the base of the glands using synchrotron radiation

**Fig. 4:**
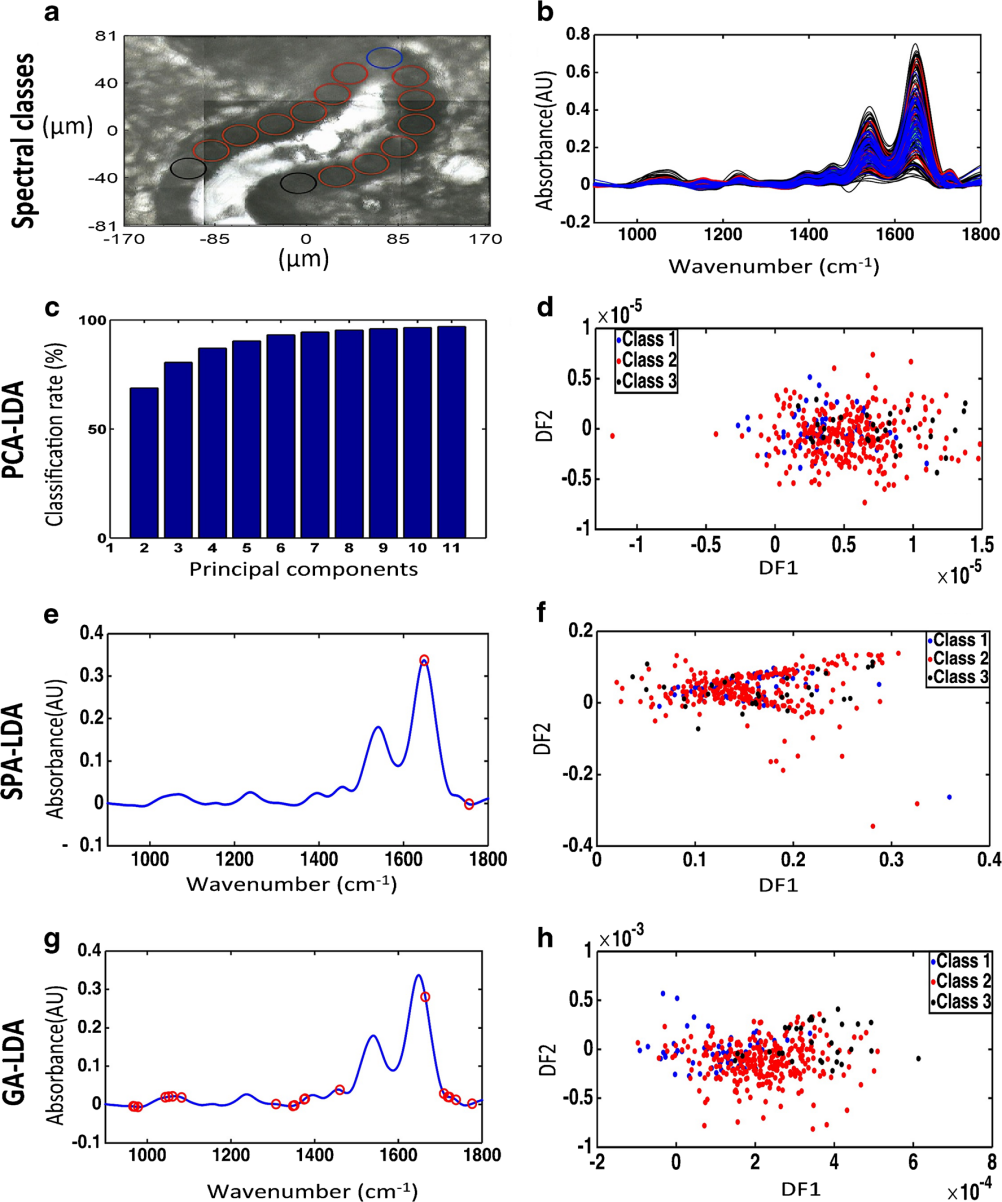
Classification of epithelial cells according to their position in the endometrial crypt base by synchrotron radiation spectral analysis using PCA-LDA, SPA-LDA and GA-LDA on FPA-FTIR microscopy-derived spectral data. **a** Micrograph with circles representing the regions sampled (this is a representation as the areas analysed were selected at different magnifications to reveal the synchrotron radiation point spectral area). **b** Spectra for the three area classes in the basalis regions of the three specimens. **c** Cost/function plot identifying the optimal number of PCs to be used for PCA. **d** Scores plot graphically representing classification by PCA-LDA. The *x*-axis represents DF1 and the *y-*axis DF2. **e** Wavenumber selection for SPA-LDA. **f** Scores plot graphically representing classification by SPA-LDA. The *x*-axis represents DF1 and the *y*-axis DF2. **g** Wavenumber selection for GA-LDA. **h** Scores plot graphically representing classification by GA-LDA. The *x*-axis represents DF1 and the *y*-axis DF2

The spatial resolution of synchrotron-based FTIR microspectroscopy is in the region of 10 μm × 10 μm. The high resolution allowed the investigation of differences that may exist between epithelial cells that reside close to the base of the glands within the basalis layer. A micrograph of the involved areas with overlaid markers was used for identification of the specific regions of which spectra were acquired. Spectra were acquired along glands, which were cut longitudinally ensuring that the deepest portion of the gland was examined (Fig. 4a). The derived spectra were divided into three classes according to their position (Fig. 4b). Class 1 included spectra taken closer to the deeper margins of the glands with the other classes being sequentially more superficial. Class 1 contained 60 spectra, class 2 contained 310 spectra and class 3 contained 30 spectra. PCA-LDA, SPA-LDA and GA-LDA of the three classes were employed using 2 wavenumbers for SPA and 20 for GA (Fig. 4c, e, g). Initial attempts to classification of these classes were underpowered as revealed by the 2-D scores plots in Fig. 4d, f and h, where segregation of classes is not readily visible, despite noticeable progression of the scores especially in the GA-LDA-derived plot.

**Fig. 5:**
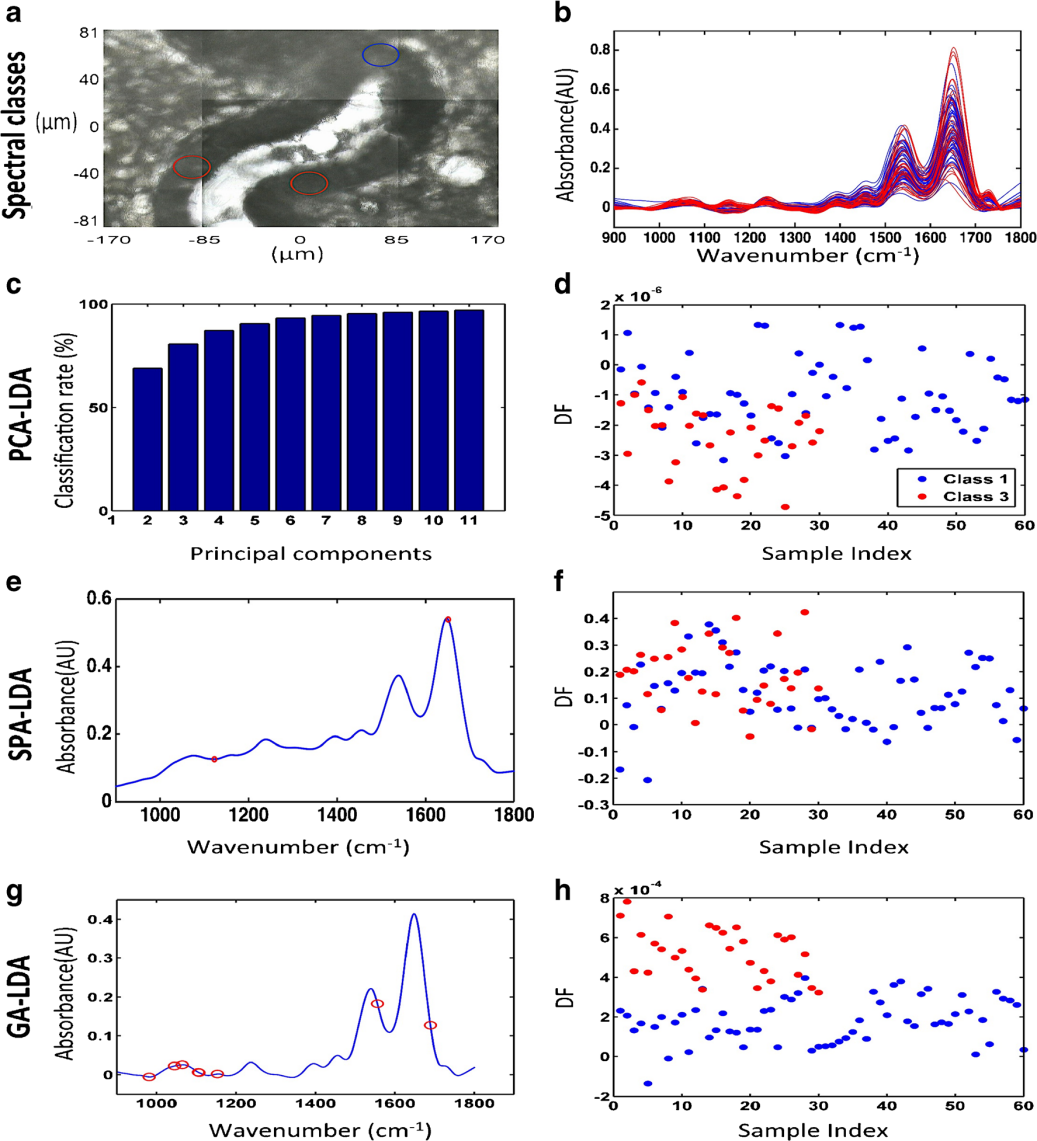
Classification of epithelial cell classes furthest apart in the endometrial crypt base by synchrotron radiation spectral analysis using PCA-LDA, SPA-LDA and GA-LDA on FPA-FTIR microspectroscopy-derived data. **a** Micrograph with circles representing the regions sampled (this is a representation as the areas analysed were selected at different magnifications to reveal the synchrotron point spectral area). **b** Pre-processed spectra of the classes furthest apart in crypt bases. **c** Cost/function plot identifying the optimal number of PCs to be used for PCA. **d** Scores plot graphically representing classification by PCA-LDA. The *x*-axis represents the sample index and the *y*-axis DF1. **e** Wavenumber selection for SPA-LDA. **f** Scores plot graphically representing classification by SPA-LDA. The *x*-axis represents the sample index and the *y*-axis DF1. **g** Wavenumber selection for GA-LDA. **h** Scores plot graphically representing classification by GA-LDA. The *x*-axis represents the sample index and the *y*-axis DF1

To identify whether there is any variation between the epithelial cells resting at the terminal end of the glands, the same analysis was employed comparing the spectra derived from the classes from above that were furthest apart (classes 1 and 3) (Fig. 5a, b). Interestingly, there was separation between these two classes when compared by all three chemometric techniques as evidenced by the 2-D scores plots in Fig. 5d, f and h, especially using GA-LDA. In this case, eight PCs (explained variance = 93.72%) were used for PCA-LDA and two wavenumbers were utilised for SPA-LDA and eight for GA-LDA (Fig. 5c, e, g and ESM Table S8), being predominantly close to the region of ~ 1234, ~ 1550 and ~ 1650 cm^−1^ which corresponds to the biomarker responses from amide III, amide II and amide I, respectively [26]. The classification rates for each technique in this case are presented in ESM Table S9.

### Synchrotron radiation-based IR spectra for the identification of the location of stem cells

**Fig. 6:**
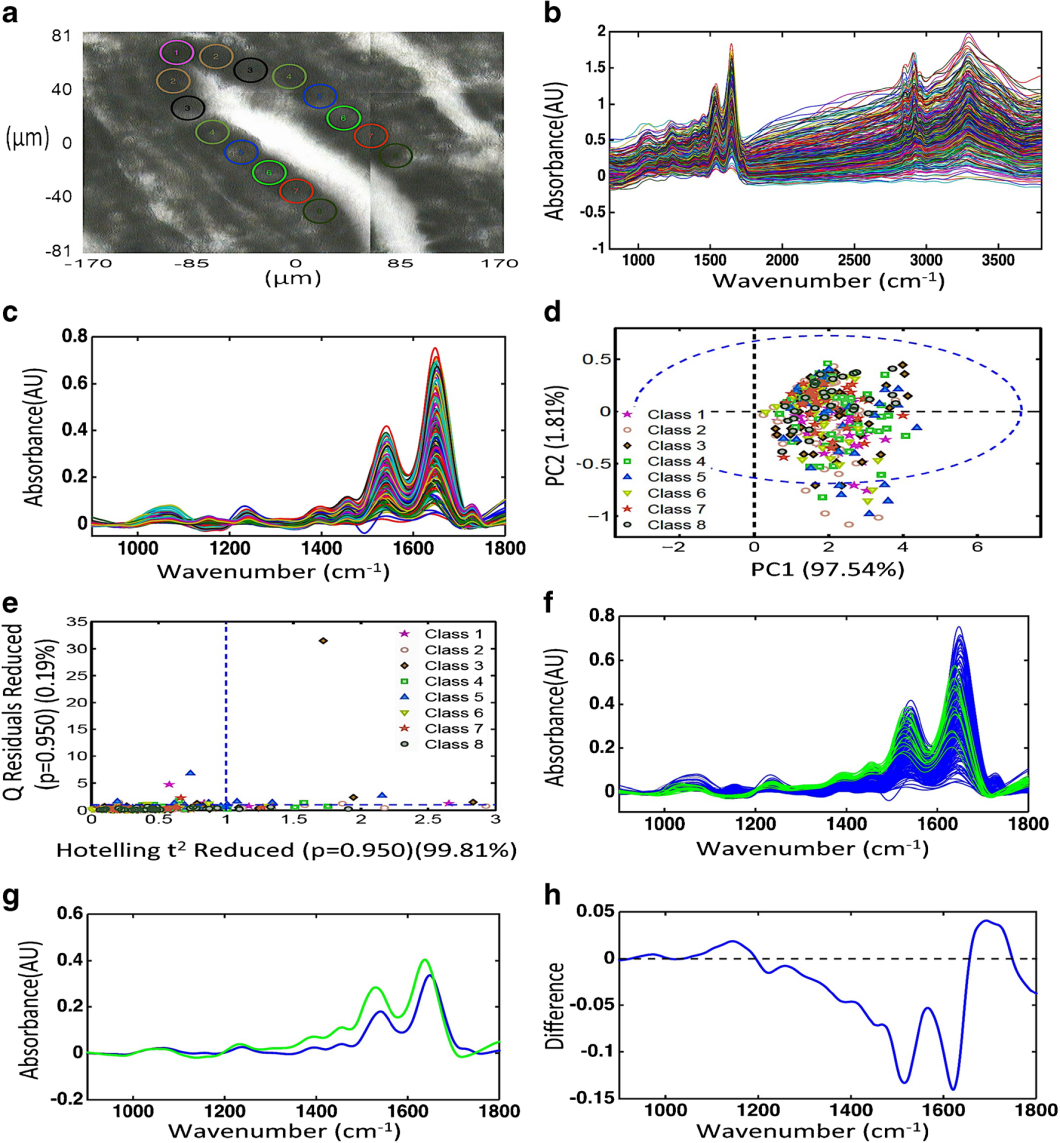
Exploratory analysis of synchrotron radiation-derived data from crypt bases to isolate putative stem cells and estimate their location. **a** Micrograph with circles representing the regions sampled (this is a representation as the regions analysed were selected at different magnifications to reveal the synchrotron radiation point spectral area). **b** Raw spectra of the classes (regions) sampled in crypt bases. **c** Pre-processed spectra of the classes (regions) sampled in crypt bases. **d** Scores plot graphically representing classification by PCA. The *x*-axis represents PC1 and the *y*-axis PC2, the dotted ellipse the 95 confidence interval (CI). **e** Scores plot graphically representing classification by Q residuals. The horizontal dotted line represents 2 standard deviations. **f** Pre-processed spectra of the epithelial cells (blue) and “outliers” (green) sampled in crypt bases. **g** Averaged spectra of the epithelial cells (blue) and “outliers” (green) sampled in crypt bases. **h** Loadings curve to identify wavenumbers responsible for segregation of “outliers” from other epithelial cells. The peaks and troughs furthest from the dotted line are the most responsible wavenumber regions

To investigate the existence of putative stem cells within the deepest/terminal portion of the glands, we searched for the existence of spectral discriminating factors between all epithelial cells in this area. Each spectrum was treated individually, but spectra were also placed into classes (1 to 8) depending on their distance from the deepest part of the gland (Fig. 6a). Ten adjacent spectra were sequentially placed in each class. The reason for this was to attempt the localisation of putative stem cells in addition to their existence. The original spectra (Fig. 6b) were cut to only include the biological fingerprint region and were pre-processed as before (Fig. 6c). They underwent exploratory PCA using the first 3 PCs (explained variance = 99.61%). Two-dimensional scores plots were extracted representing the first 2 PCs (Fig. 6d). Each point on the resulting scores plot represented a single point on the image maps. Using a 95% confidence interval (CI), spectral points that segregated from the clustered spectra were identified. These “outliers” were particularly obvious on the PC2 axis and included mostly cells from classes 1, 2, 5 and 6. Interestingly, when Q residual analysis was applied, classes 1 and 5 also featured outlier spectra (Fig. 6e). Following analysis by these unsupervised techniques, spectra were classified depending on whether they were identified as outliers or not (Fig. 6f). The averaged spectra resulting from these two classes (“normal” and “outliers”) exposed visible differences (Fig. 6g). A resulting subtraction spectrum identified differences in the absorption bands near 1080 cm^−1^ (symmetric PO_2_^−^ stretching vibrations in RNA and DNA), 1390 cm^−1^ (nucleic acids), 1550 cm^−1^ (amide II) and 1650 cm^−1^ (amide I) (Fig. 6h).

**Fig. 7:**
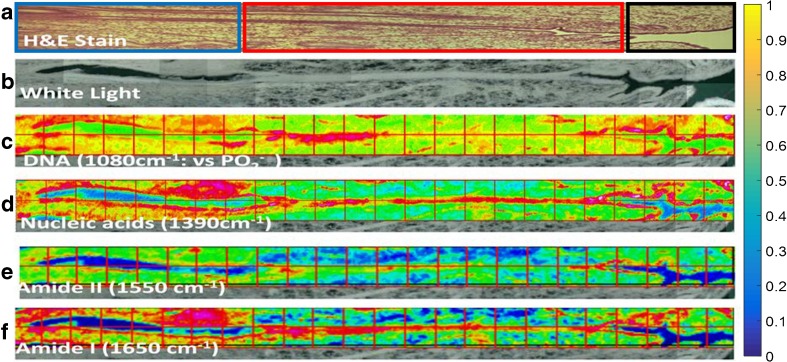
Intensity images based on FPA imaging (pink represents highest intensity while blue the lowest). **a** H&E-stained longitudinally cut uterine glandular crypt (circled in blue: cells in the deepest portion of the gland; circled in red: basalis/functionalis cells; circled in black: cells close to the gland opening into the endometrial cavity). **b** White light image of the longitudinally cut uterine glandular crypt (magnification ×15). **c** 2-D intensity map derived following FPA spectral analysis at the wavenumber 1080 cm^−1^ overlaid onto a white light micrograph (magnification ×15). **d** 2-D intensity map derived following FPA spectral analysis at the wavenumber 1390 cm^−1^ overlaid onto a white light micrograph (magnification ×15). **e** 2-D intensity map derived following FPA spectral analysis at the wavenumber 1550 cm^−1^ overlaid onto a white light micrograph (magnification ×15). **f** 2-D intensity map derived following FPA spectral analysis at the wavenumber 1650 cm^−1^ overlaid onto a white light micrograph (magnification ×15). Colour bar: normalised absorbance for **c**–**f**

These absorption bands were utilised to produce intensity images based on the FPA spectra to show where in the gland their intensity is maximal to attempt visualisation of “outlier” cellular regions that include putative stem cells (Fig. 7). Although the definition of the FPA images does not allow precise localisation of the highest intensities, it is broadly evident that there are in general three areas within the gland where these outlier cells reside: these are along the deepest portion of the gland (Fig. 7a—circle in blue), the basalis/functionalis junction (Fig. 7a—circled in red) and close to the most superficial part of the gland opening into the endometrial cavity (Fig. 7a—circled in black). The stem cells were found within the population of epithelial cells lining the basalis portion of endometrial glands.

## Discussion

This study employed powerful chemometric techniques in conjunction with synchrotron radiation and globar IR technologies to classify epithelial histological areas within the endometrial glands. It also explored the potential of such technologies in isolating putative stem cells and determining their location within the endometrial epithelium. Since the progress in characterising the human endometrial epithelial stem cells has been particularly poor, our method may attract the attention of endometrial biologists.

Pathophysiological, morphological and molecular alterations of epithelial cells that line the glands in the basalis and functionalis layers may be associated with both benign and malignant diseases arising from the endometrium. Therefore, understanding the differences in molecular composition of the cells within the examined histological areas may assist directing research to further pinpoint the exact locations of such pathological changes and to potentially formulate curative strategies.

Computational analysis of the obtained spectra identified significant differences between the epithelial cells lining the glands in the basalis and functionalis regions. Some of the discriminating wavenumbers for this variation remained constant when uterine specimens from different individuals were examined separately. Namely, amide I and stretching of C=N bonds correctly classified the two histological regions. This molecular variability between the two layers may assist with the further characterisation of the basalis epithelial cells, which are postulated to give rise to many endometrial proliferative conditions such as endometriosis. The functionalis layer is shed during the menstrual shedding leaving the basalis from which the new functionalis is thought to be regenerated. Noe et al. [3] have suggested that the cells from the basalis layer are responsible for the establishment of ectopic endometriotic lesions and that it is further supported by the baboon model of induction of endometriosis [17]. In the baboons, the endometrium biopsied during day 2 of the menses (when the basalis is easily accessible) when instilled in the pelvic cavity gives rise to identical endometriotic deposits to humans. This is likely to be due to the enrichment of stem cells in the basalis layer, that is sampled during menses, and these cells are likely to survive and establish endometriotic lesions. Therefore, identifying such discriminatory spectroscopic biomarkers can also be significant due to their potential for translation into clinical practice. Biomarkers identified using the above techniques may be further explored to interrogate the basalis endometrial layer of women with and without endometriosis.

Several studies have attempted the identification of adult stem cells within the basalis layer [1, 6]. These studies have utilised different stem cell-specific activities for identification [9, 10, 13]. Our study intended to isolate and locate putative epithelial stem cells using morphological, molecular and chemical variability that exists within the cells lining the glandular glands. There is expanding evidence that FTIR microspectroscopy is capable of identifying stem cells in several tissues including the cornea, epidermis and intestine [23, 37–40]. The chemometric analysis of the IR data we acquired was able to identify specific putative stem cell locations within the population of epithelial cells. Both FPA and synchrotron radiation-based techniques identified putative stem cells at the deepest part of the gland and at a distance of about 50 μm from that location. This indicates that there are at least two sets of stem cells residing in these gland bases. We speculate that the stem cells have different functions: one being dormant and the other actively differentiating to more specialised daughter cells.

Analysis of the synchrotron radiation data has also shown mild progressive differences between the epithelial cells lining the bases of the glands. Although when the synchrotron radiation-derived spectral data from these cells were compared by dividing them into three classes, their variability was minimal; when comparing the classes positioned furthest apart, they exhibited significant differences. This points to a progressive alteration in the molecular structure of epithelial cells even in the basalis layer.

All samples in our study were obtained from women that had hysterectomies for reasons other than endometrial pathology or malignancy. Our aim was to perform preliminary spectroscopic investigations on these tissues to evaluate the performance of these techniques in identifying different cellular lineages within the endometrial glands. The variability between endometrial samples depends on cycle phase, hormonal factors, lifestyle, body mass index, weight, diet and alcohol consumption, parity and several other factors; therefore, obtaining completely homogeneous endometrial samples from different individuals is virtually impossible. Although our sample size was small, all three women included were sampled within the secretory phase of the cycle to remove cycle-dependent variability and all women had histologically confirmed a healthy endometrium without any prior hormonal treatments. The three different samples in our study were used to support information derived when using all specimens together.

Identification of endometrial adult stem cells may be of value in all endometrial proliferative disease including carcinogenesis research as they may be involved in related processes by their alteration into cancer stem cells [15]. Their localisation by means of specific biomarker as extracted by spectroscopic techniques may in the future provide diagnostic and therapeutic avenues for uterine cancer.

## Conclusion

With this study, we demonstrate that SR-FTIR microspectroscopy coupled with multivariate computational analysis and wavenumber selection techniques might be able to identify putative stem cells within the population of epithelial cells lining the basalis portion of endometrial glands. Specific identification of the location of these cells remains elusive, but potential stem cells were segregated from their surrounding cells based on their inherent IR spectroscopic signatures. Specific spectral biomarkers responsible for this segregation were identified and corresponded mainly to PO_2_^−^ vibrational modes of DNA and RNA, nucleic acids and amides I and II. In addition, FPA-FTIR spectroscopy coupled with similar chemometric analysis was able to consistently differentiate the epithelial cells lining the basalis portion of the endometrial glands from those lining the functionalis portion. Further future developments in both spectroscopic technologies and related chemometric techniques may be able to track cellular lineages and individual cells within all tissues, therefore potentially revealing physiological and pathological tissue functions based on morphological alterations.

## Electronic supplementary material

ESM 1 (PDF 2.92 mb)

## Abbreviations

DF: Discriminant function
DNA: Deoxyribonucleic acid
FN: False negative
FP: False positive
FPA: Focal plane array
FPA-FTIR: Focal plane array-based Fourier-transform infrared
FTIR: Fourier-transform infrared
GA: Genetic algorithm
GA-LDA: Genetic algorithm–linear discriminant analysis
H&E: Haematoxylin and eosin
IR: Infrared
KS: Kennard-Stone
LDA: Linear discriminant analysis
MCT: Mercury cadmium telluride
NA: Numerical aperture
PCA: Principal component analysis
PCA-LDA: Principal component analysis–linear discriminant analysis
PCs: Principal components
RNA: Ribonucleic acid
SNR: Signal-to-noise ratio
SPA: Successive projections algorithm
SPA-LDA: Successive projections algorithm–linear discriminant analysis
SR-FTIR: Synchrotron radiation-based Fourier-transform infrared
TN: True negative
TP: True positive
