# Supplementary material for: Synchrotron- and focal plane array-based Fourier-transform infrared spectroscopy differentiates the basalis and functionalis epithelial endometrial regions and identifies putative stem cell regions of human endometrial glands
Source: Anal Bioanal Chem. 2018 May 9;410(18):4541–54. doi: 10.1007/s00216-018-1111-x (PMC6021468; doi:10.1007/s00216-018-1111-x)
Supplement: Supplementary file 1 — (PDF 2.92 mb) [file 216_2018_1111_MOESM1_ESM.pdf]

Analytical and Bioanalytical Chemistry

Electronic Supplementary Material

Synchrotron- and focal plane array-based Fourier-transform infrared spectroscopy differentiates the basalis and functionalis epithelial endometrial regions and identifies putative stem cell regions of human endometrial glands

Georgios Theophilou, Camilo L. M. Morais, Diane E. Halliwell, Kássio M. G. Lima, Josephine Drury, Pierre L. Martin-Hirsch, Helen F. Stringfellow, Dharani K. Hapangama, Francis L. Martin

Table S1 Number of averaged spectra arranged by area as selected on slides for FPA analysis

Basalis		*Functionalis*	
Sampled area code	Number of averaged spectra	Sampled area code	Number of averaged spectra
1	2140	1	5127
2	510	2	945
3	1324	3	1534
4	2278	4	1589
5	3448	5	1534
6	4647	6	2064
7	1823	7	1299
8	1753	8	1100
9	1398	9	2060
10	1450	10	3876
11	1563	11	2155
12	803	12	1517
13	1906	13	3230
14	592	14	2525
15	1894	15	13211
16	803	16	10123
17	8195	17	7143
18	1830	18	4800
TOTAL	38357	TOTAL	65832

Table S2 Selected variables by each chemometric technique employed for the differentiation of *basalis* from *functionalis* epithelial regions using FPA spectral data

	Selected variables as wavenumbers (cm^{-1})
SPA-LDA	1236, 1506, 1519, 1552, 1566, 1616, 1627, 1654, 1666, 1672, 1687, 1693
GA-LDA	923, 929, 950, 1014, 1029, 1033, 1072, 1097, 1411, 1510, 1602, 1662, 1666, 1746

Table S3 Overall classification rates by each chemometric technique employed to differentiate *basalis* from *functionalis* epithelial regions using FPA spectral data

	PCA-LDA	SPA-LDA	GA-LDA
Classification rate for calibration set (%)	66.7	87.5	95.8
Classification rate for validation set (%)	50.0	100.0	100.0
Classification rate for prediction set (%)	33.3	100.0	100.0

Table S4 Overall classification rates by each chemometric technique employed to differentiate of *basalis* from *functionalis* tissues for individual specimen sets using FPA spectral data

	PCA-LDA	SPA-LDA	GA-LDA
**SPCN 1**			
Classification rate for calibration set (%)	100.0	100.0	100.0
Classification rate for validation set (%)	66.7	100.0	100.0
Classification rate for prediction set (%)	100.0	75.0	50.0
**SPCN 2**			
Classification rate for calibration set (%)	100.0	100.0	100.0
Classification rate for validation set (%)	0.0	100.0	100.0
Classification rate for prediction set (%)	100.0	100.0	100.0
**SPCN 3**			
Classification rate for calibration set (%)	93.3	100.0	93.3
Classification rate for validation set (%)	100.0	100.0	100.0
Classification rate for prediction set (%)	100.0	66.7	66.7

Table S5 Selected variables by each chemometric technique employed to differentiate *basalis* from *functionalis* epithelial regions for individual specimen sets using FPA spectral data

	Selected variables as wavenumbers (cm^{-1})
**SPCN 1**	
SPA-LDA	1496, 1554, 1567, 1602, 1618, 1627, 1662, 1681, 1685, 1691, 1695
GA-LDA	991, 1083, 1134, 1243, 1326, 1429, 1458, 1554, 1604, 1616, 1629, 1714
**SPCN 2**	
SPA-LDA	975, 1238, 1633, 1683
GA-LDA	973, 1251, 1616, 1620, 1658
**SPCN 3**	
SPA-LDA	1083, 1220, 1236, 1510, 1548, 1556, 1610, 1623, 1625, 1631, 1660, 1687, 1693
GA-LDA	933, 1139, 1297, 1299, 1747

Table S6 Overall classification rates for each chemometric technique employed to identify inter-individual variation between specimens within *basalis* epithelial layer

	PCA-LDA	SPA-LDA	GA-LDA
Classification rate for calibration set (%)	93.3	100.0	100.0
Classification rate for validation set (%)	75.0	100.0	100.0
Classification rate for prediction set (%)	100.0	100.0	75.0

Table S7 Selected variables used for each chemometric technique employed to identify inter-individual variability within the *basalis* layer using FPA spectral data

	Selected variables as wavenumbers (cm^{-1})
SPA-LDA	1236, 1548, 1564, 1585, 1602, 1614, 1633, 1648, 1666, 1683, 1689, 1743
GA-LDA	900, 983, 991, 1016, 1091, 1122, 1137, 1182, 1203, 1681, 1753

Table S8 Selected variables used for each chemometric technique employed to differentiate Class 1 and Class 3 regions at the epithelial crypt bases using synchrotron data

	Selected variables as wavenumbers (cm^{-1})
SPA-LDA	1265, 1647
GA-LDA	981, 1045, 1064, 1105, 1107, 1153, 1556, 1689

Table S9 Overall classification rates by each chemometric technique employed to differentiate Class 1 and Class 3 regions at the epithelial crypt bases using synchrotron data

	PCA-LDA	SPA-LDA	GA-LDA
Classification rate for calibration set (%)	74.6	58.7	96.8
Classification rate for validation set (%)	69.2	69.2	100.0
Classification rate for prediction set (%)	78.6	50.0	92.9

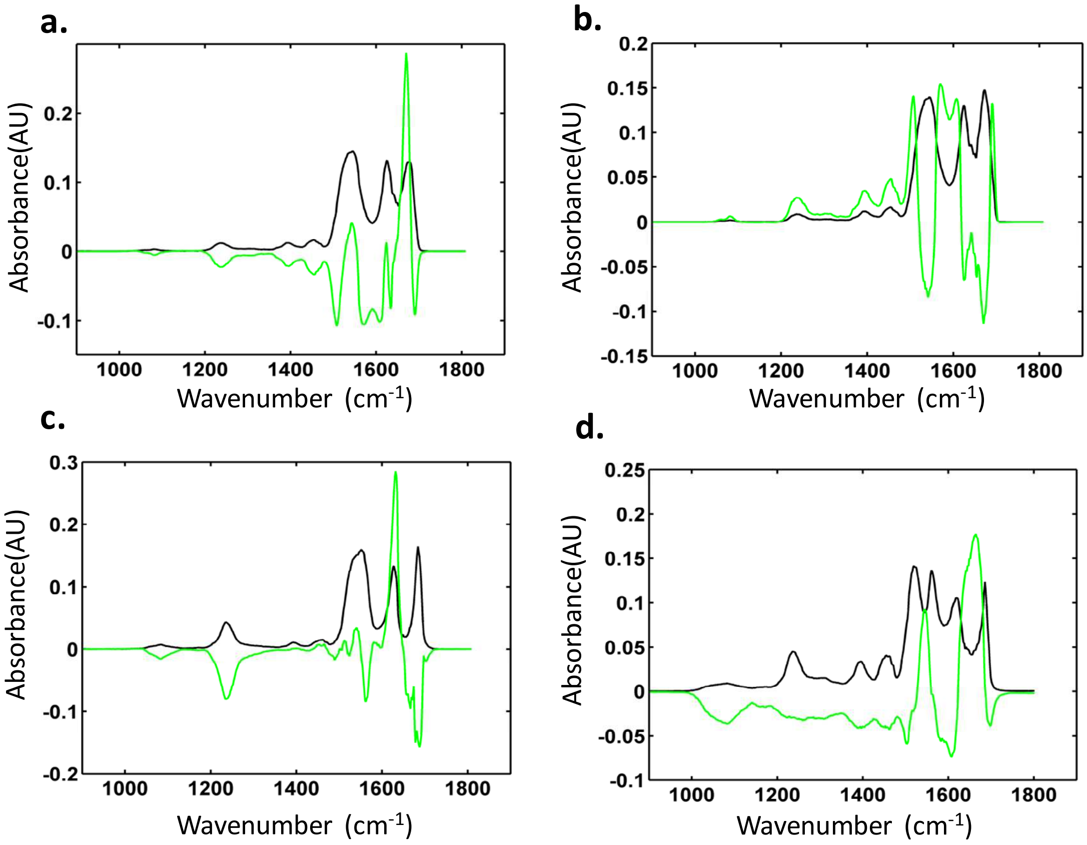

Fig. S1 Loadings curves derived from PCA-LDA for the comparison of basalis and functionalis epithelial regions using FPA spectroscopy; (Black line: Loadings on PC1; green line: Loadings on PC). a. Loadings curve for *basalis* and *functionalis* comparison for all specimens, b. Loadings curve for *basalis* and *functionalis* comparison for SPCN 1, c. Loadings curve for *basalis* and *functionalis* comparison for SPCN 2, d. Loadings curve for *basalis* and *functionalis* comparison for SPCN 3

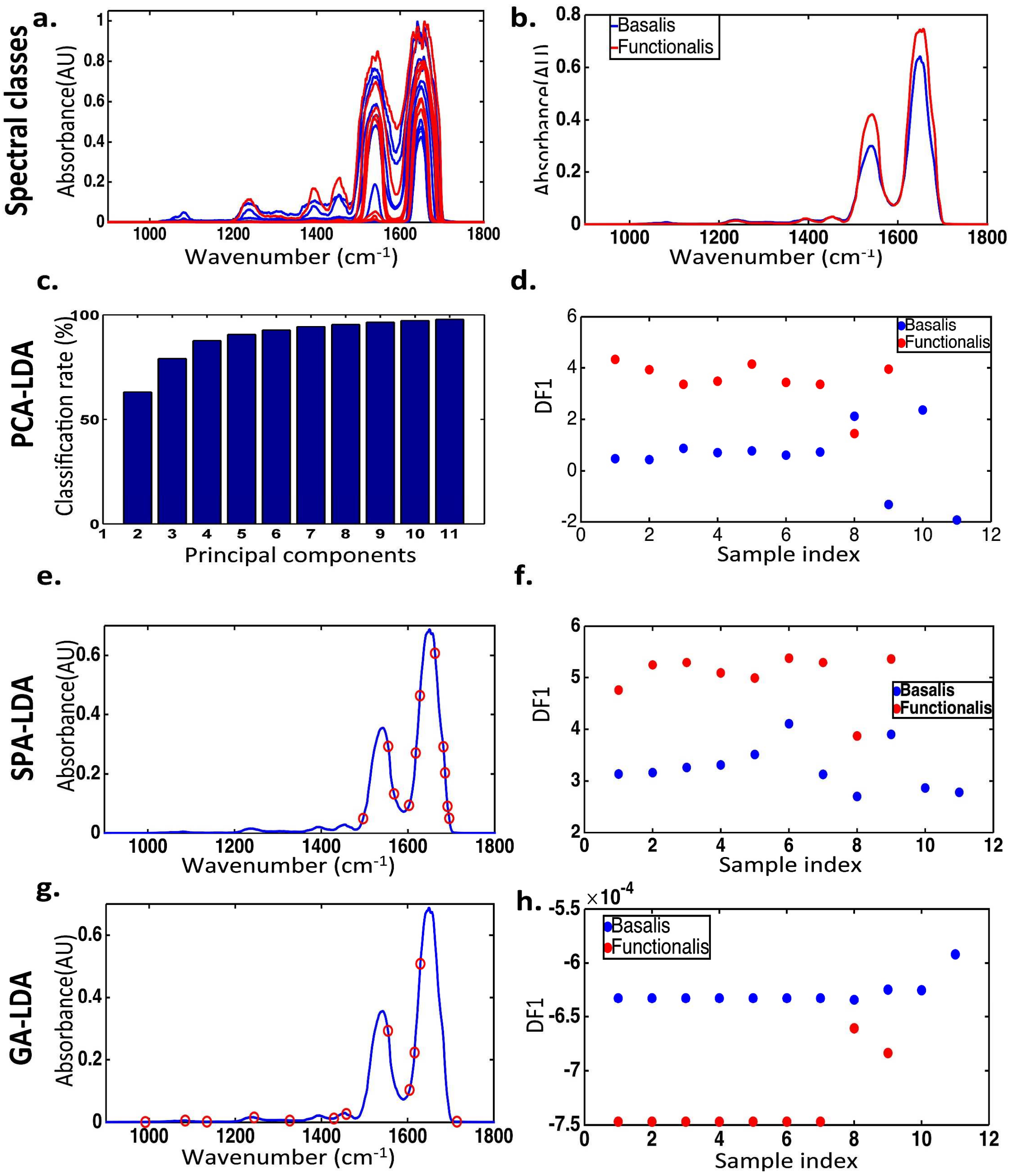

Fig. S2 Classification of *Basalis* and *Functionalis* regions by spectral analysis using PCA-LDA, SPA-LDA and GA-LDA on FPA-FTIR derived data. (red= *Functionalis*, blue= *Basalis*): SPCN 1. a. Pre-processed spectra containing the biological fingerprint spectral regions, b. Averaged spectra for the *basalis* and *functionalis* regions, c. Cost/ function plot identifying the optimal number of PCs to be used for PCA, d. Scores plot graphically representing classification by PCA-LDA. The *x*-axis represents the sample index and the *y*-axis DF1, e. Wavenumber selection for SPA-LDA, f. Scores plot graphically representing classification by SPA-LDA. The *x*-axis represents the sample index and the *y*-axis DF1, g.

Wavenumber selection for GA-LDA, h. Scores plot graphically representing classification by GA-LDA. The *x*-axis represents sample index and the *y*-axis DF1

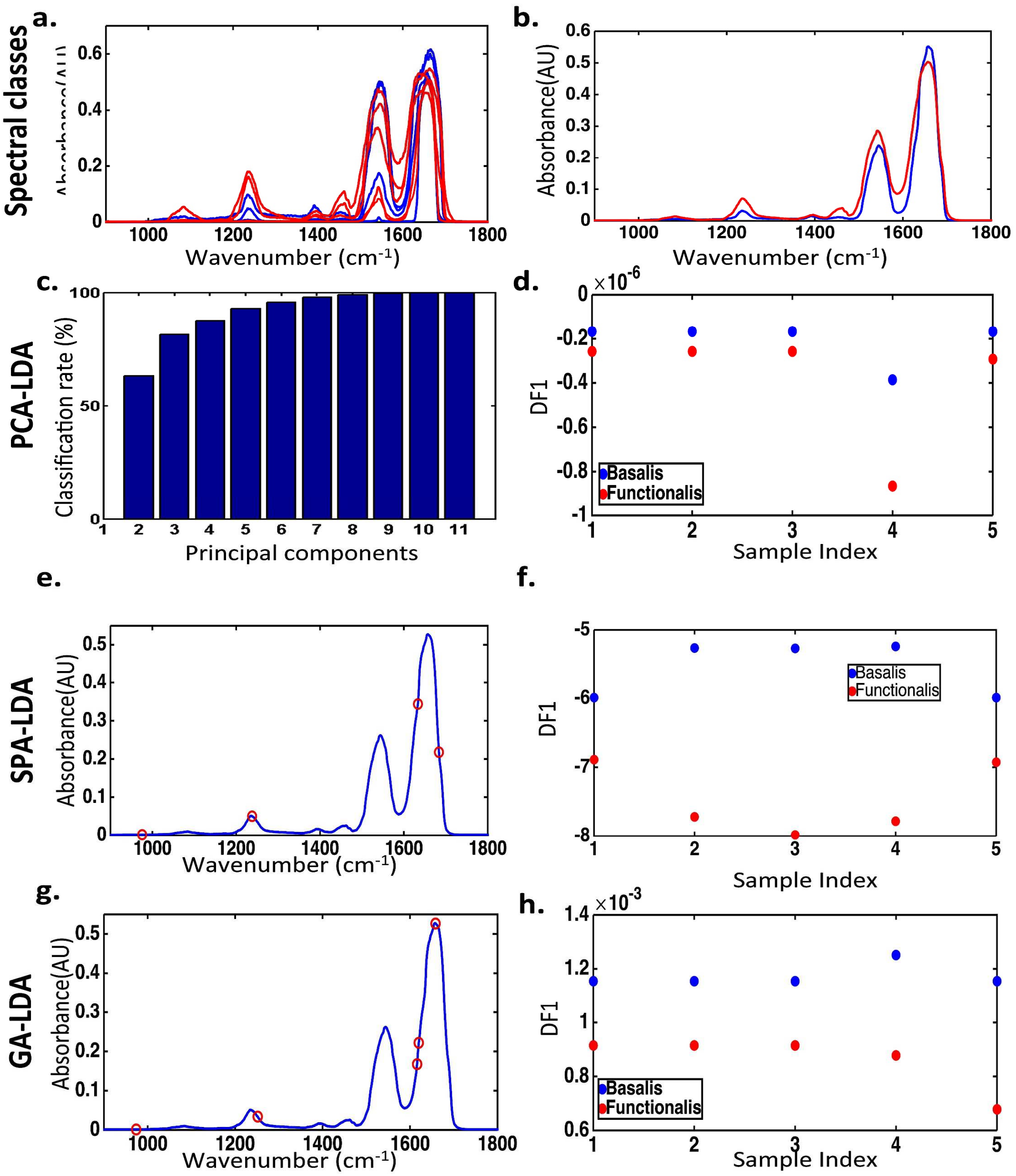

Fig. S3 Classification of *Basalis* and *Functionalis* regions by spectral analysis using PCA-LDA, SPA-LDA and GA-LDA on FPA-FTIR derived data. (red= *Functionalis*, blue= *Basalis*): SPCN2. a. Pre-processed spectra containing the biological fingerprint spectral regions, b. Averaged spectra for the *basalis* and *functionalis* regions, c. Cost/ function plot identifying the optimal number of PCs to be used for PCA, d. Scores plot graphically representing classification by PCA-LDA. The *x*-axis represents the sample index and the *y*-axis DF1, e. Wavenumber selection for SPA-LDA, f. Scores plot graphically representing classification by SPA-LDA. The *x*-axis represents the sample index and the *y*-axis DF1, g. Wavenumber selection for GA-LDA, h. Scores plot graphically representing classification by GA-LDA. The *x*-axis represents sample index and the *y*-axis DF1

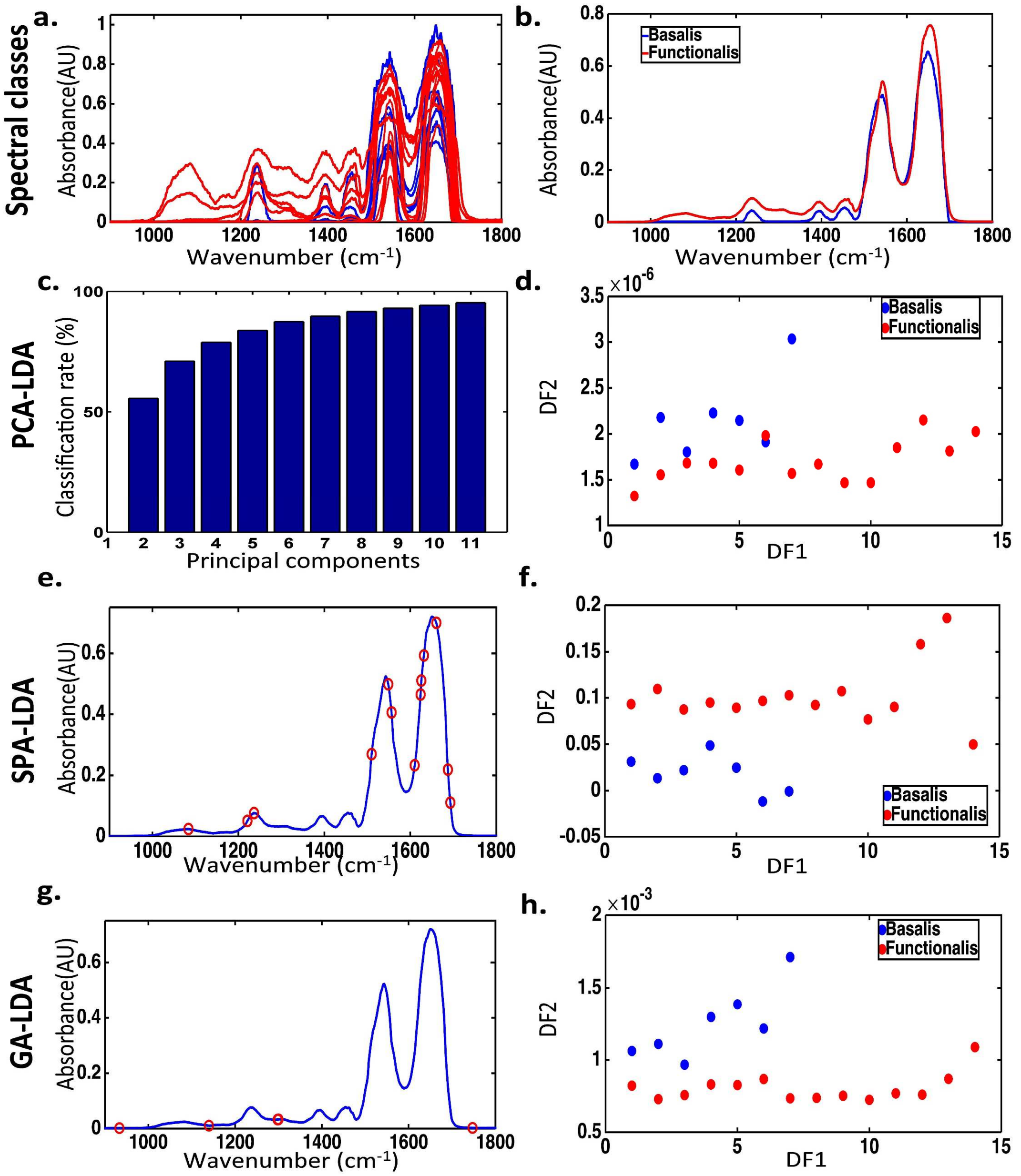

Fig. S4 Classification of *Basalis* and *Functionalis* regions by spectral analysis using PCA-LDA, SPA-LDA and GA-LDA on FPA-FTIR derived data. (red= *Functionalis*, blue= *Basalis*): SPCN 3. a. Pre-processed spectra containing the biological fingerprint spectral regions, b. Averaged spectra for the *basalis* and *functionalis* regions, c. Cost/ function plot identifying the optimal number of PCs to be used for PCA, d. Scores plot graphically representing classification by PCA-LDA. The *x*-axis represents the sample index and the *y*-axis DF1, e. Wavenumber selection for SPA-LDA, f. Scores plot graphically representing classification by SPA-LDA. The *x*-axis represents the sample index and the *y*-axis DF1, g. Wavenumber selection for GA-LDA, h. Scores plot graphically representing classification by GA-LDA. The *x*-axis represents sample index and the *y*-axis DF1

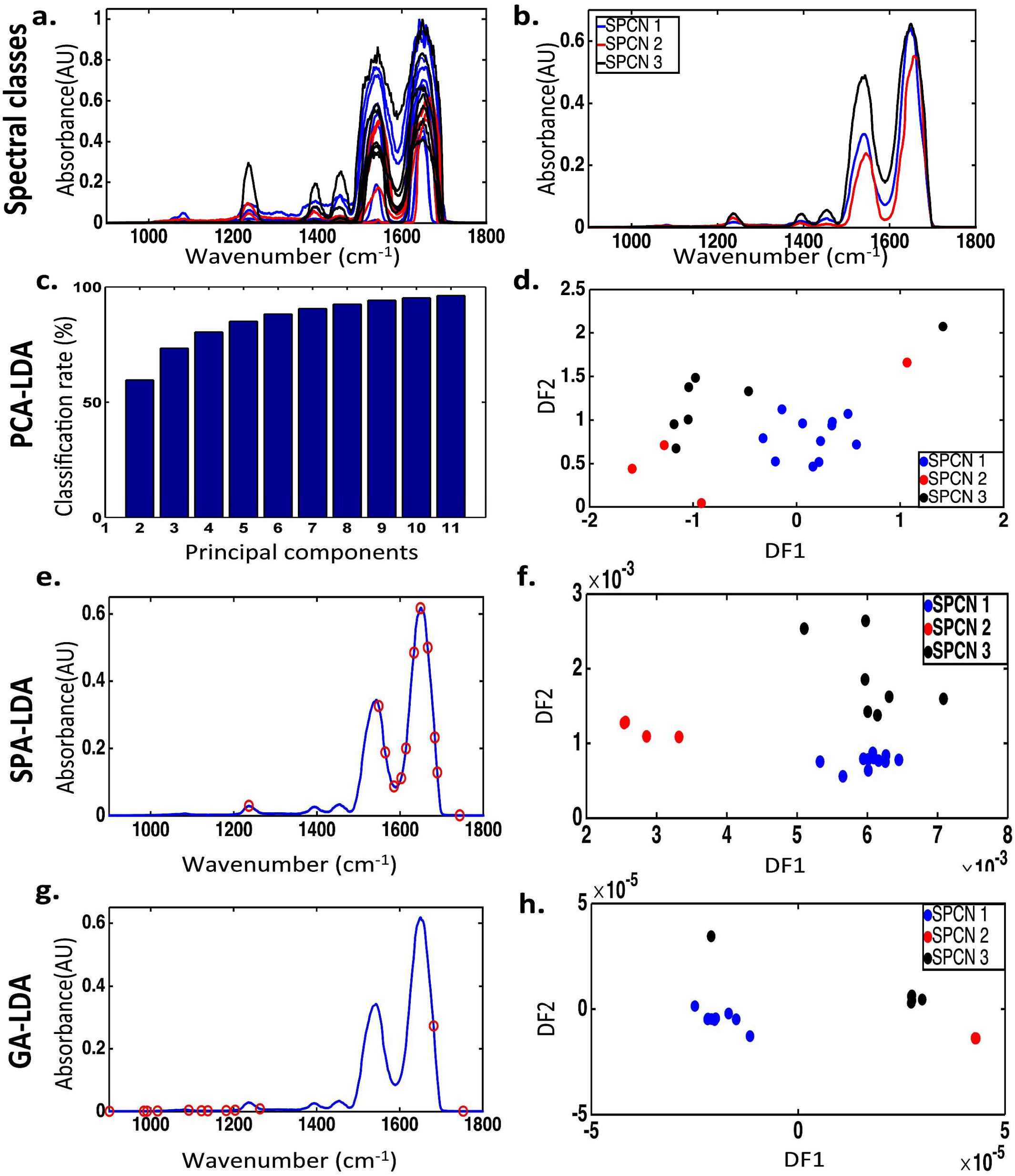

Fig. S5 Classification specimens based on variability of their *Basalis* regions by spectral analysis using PCA-LDA, SPA-LDA and GA-LDA on FPA-FTIR derived data. (blue= SPCN1, red= SPCN2, black, SPCN3). a. Pre-processed spectra containing the biological fingerprint spectral regions, b. Averaged spectra for the *basalis* regions of the three specimens, c. Cost/ function plot identifying the optimal number of PCs to be used for PCA, d. Scores plot graphically representing classification by PCA-LDA. The *x*-axis represents DF1 and the *y*-axis DF2, e. Wavenumber selection for SPA-LDA, f. Scores plot graphically representing classification by SPA-LDA. The *x*-axis represents DF1 and the *y*-axis DF2, g. Wavenumber selection for GA-LDA, h. Scores plot graphically representing classification by GA-LDA. The *x*-axis represents DF1 and the *y*-axis DF2
